# Unpacking Relational Dignity: In Pursuit of an Ethic of Care for Outdoor Therapies

**DOI:** 10.3389/fpsyg.2022.766283

**Published:** 2022-02-10

**Authors:** Nevin J. Harper, Carina Ribe Fernee

**Affiliations:** ^1^Faculty of Human and Social Development, University of Victoria, Victoria, BC, Canada; ^2^Department of Child and Adolescent Mental Health, Sørlandet Hospital HE, Kristiansand, Norway; ^3^Faculty of Health and Sport Sciences, University of Agder, Kristiansand, Norway

**Keywords:** outdoor therapy, relational dignity, ethic of care, ecological dignity, wilderness therapy, dignity

## Abstract

Dignity is a universal principle that requires us to treat every person as having worth beyond who a particular person is or what they do. Dignity is a complex and sometimes contested idea, that at times can be compromised in health care and allegedly also within the practice of outdoor therapy. Outdoor therapies comprise a range of therapeutic approaches including nature-based therapy, adventure therapy, animal-assisted therapy, forest therapy, wilderness therapy, surf therapy, and more. Within the literature of outdoor therapies there has been limited research on ethics related to common understandings of care concepts such as relational dignity and human rights. The aim of this paper is therefore to unravel briefly whether dignity in general, and relational qualities of dignified care more particularly, might be a useful concept to apply in order to support an ethical practice in outdoor therapies.

## Introduction

“The unconditional and non-descriptive character of dignity as a principle is both its force and its weakness; it appeals to many and nobody could be against it, but it refers to different values in particular situations. Tensions in practice emerge when there are differences in how to understand a respectful approach, or how to best interpret dignity” (Pols et al., 2018, p. 91).

Dignity is a foundational principle, referring to a human right for all people. The ethical principle is that all humans *have* dignity, simply because they are human (Pols et al., 2018). Protecting this *intrinsic* (Leget, 2013) or *inherent* (Nordenfelt, 2004) dignity would mean to protect humans and humanity itself. According to Pols et al. (2018), this requires us to treat every person as having a dignity or worth beyond the worth of who a particular person *is* or what they *do*. When dignity is seen as inherent worth, it is something that cannot be acquired, but can be violated, and so-called indignities are affronts to human dignity. However, to describe situations where this happens, the understanding of the somewhat abstract principle needs to be made concrete, beyond an appeal for care that is worthy or humane.

Dignity remains a complex and sometimes contested concept, and when loss of dignity is reported within our field of practice, it is our ethical obligation as practitioners and researchers, to respond and to ensure that we protect and restore dignity as best we can. Successful therapy relies on the quality of relationship between therapist and client (alliance) and is one of the strongest in-treatment factors influencing positive outcomes (Elvins and Green, 2008; Wampold, 2021). Relational dignity carries significant implications beyond ethical therapeutic practice; dignity has been described as the concept that ties health and human rights together (Jacobson, 2009). Within the literature of outdoor therapies there has been a lack of dialog and research on ethics related to common understandings of care concepts such as relational dignity and human rights (Mitten, 1994; Becker, 2010; Harper, 2017). Recent research brings to light a dire need for further empirical and philosophical development of outdoor therapy ethics (Dobud, 2021; Harper et al., 2021). These recent publications provide the impetus for this article, but the desirability of relational dignity as an explicit value in the practice of outdoor therapy and healthcare has been recognized and recently called for (see Fernee and Gabrielsen, 2020). While we identify the scarcity of literature on this topic, we do acknowledge that practitioners and researchers constantly strive to improve practice and that critical discussions and dialog are present at conferences, within professional associations and trainings.

Outdoor therapies include a range of therapeutic approaches identified as nature-based therapy, adventure therapy, wilderness therapy, animal-assisted therapy, forest therapy, surf therapy and more (Harper and Dobud, 2020; Harper et al., 2021). The umbrella term of outdoor therapies captures practices sharing three common factors: (1) place-based (generally outdoors), (2) active bodily-engaged practices, and (3) place client, therapist, and nature in a relational triad thereby removing a human-nature dichotomy (Harper and Doherty, 2020). We acknowledge that “nature,” “wilderness” and other terms are contested (as colonial, romantic, reductionist) concepts and point readers to critical perspectives on outdoor therapies published elsewhere (Harper et al., 2017; Mitten, 2020). Most outdoor therapies have been found, in repeated systematic reviews, to have positive health and wellbeing benefits (see Cooley et al., 2020; Stier-Jarmer et al., 2021). Wilderness therapy is one internationally practiced form of outdoor therapy in which contextual realities of increased intensity, duration, challenge and other significant factors are present while intact groups travel in less-inhabited and more remote “wilderness” areas for multiple days to weeks (Fernee et al., 2019). These factors complicate and heighten the need for relational dignity (e.g., concerns about power differentials, reliance on the skilled leader, sense of physical and emotional safety), hence requiring careful attentiveness from the practitioners, and arguably further research, dialog and philosophical practice development (Mitten, 1994; Harper et al., 2019).

As authors, we believe it to be obvious that all clients deserve to be treated with a respect that acknowledges their personal dignity and hope for this to become an internalized norm across all forms of outdoor therapies. In this paper, we begin a conceptual and practical exploration of what relational dignity means and how it may translate into an ethic of care for psychological interventions in the outdoors. As such, the aim of this paper is to unravel briefly how dignity in general, and relational qualities of dignified care more particularly, might be understood in the literature, again as a means to consider the potential of relational dignity as a useful concept to excel and improve practice in outdoor therapies.

## Unpacking Relational Dignity

Dignity refers to the intrinsic value of each human life and has been described as nothing less than a central feature of our humanity. The United Nation’s *Universal Declaration of Human Rights* recognizes the inherent dignity and equal rights of all members of the human family as the very foundation of freedom, justice and peace in the world (Miller, 2017). These essential facets of dignity should not only be understood and ideally agreed upon, but most importantly acted on, by all healthcare practitioners and indeed, everyone. In the context of healthcare, dignity appeals to fundamental values of what good care entails (Pols et al., 2018).

However, it is often easier to conceptualize dignity in negative terms – for instance by recognizing undignified or disrespectful treatment – rather than in the positive. Second, dignity has intrinsic, relational, and distributional dimensions, that all have implications primarily for an individual’s sense of self-worth and well-being, and secondarily for a proposed ethics of care (Pringle et al., 2021). We shall return to all of these aspects of dignity after first having considered the most commonly expressed concerns regarding the concept.

### Complaints Against the Concept of Dignity

While dignity overall is a sustained and even reemerging concept across a number of disciplines, there are wide-ranging complaints against the concept that covers everything from its supposedly general uselessness to its exclusionary nature (Miller, 2017). We feel obliged to consider some of these complaints, before still choosing to operationalize dignity, and propose it as a fundamental value in care ethics for outdoor therapies.

Macklin (2003) claims that dignity is a vague reworking of other values that she finds more useful and precise, such as respect for autonomy. Another complaint along the same lines is the charge that dignity is an ineffectual concept. Miller (2017) refers to Fanon (2008) assertions that respect for human dignity alone cannot alter reality. In the context of oppression, for instance, there may not be any other option than to fight and resist, thus rendering the idea of dignity an impotent notion against systemic powers and interests. These two concerns relate to a third criticism, expressed by theorists interested in issues such as race, class, gender, and disability. These scholars rightfully direct awareness toward *who* the concept of dignity has tended to include and benefit, and on the other hand, who are then excluded from its circle? As such, voicing the concern that dignity might so happen to be an overwhelming white, Western, male, and able-bodied notion (Miller, 2017).

Let us apply the latter concern to the context of outdoor therapies. Some wilderness therapy organizations in the American for-profit sector, for example, are often inaccessible due to high costs. Additionally, forced transport is employed in some wilderness therapy programs, which includes youth being taken from their family homes against their will (Dobud, 2021). Are some parts of outdoor therapy practices undermining the autonomy and respect for its participants? Are for instance, teens who are identified as struggling and considered in need of out-of-home treatment not deserving of being treated with dignity at all times? Forced transport and resultant involuntary treatment strips autonomy and choice from youth and compromises their inalienable rights while also compromising practictioners’ adherence to their professional codes of ethics allowing potentially indignified scenarios to occur.

Another concern particularly relevant for outdoor therapy approaches is that dignity as a concept has historically excluded the more-than-human nature (e.g., forests, rivers, animals, etc.) although the animal-assisted therapies have long-provided direction on this with care and dignity afforded to therapy animals (horses, dogs, etc.). Thus in our proposed ethics of care for outdoor therapies, we include all of nature – human and non-human – as vital parts of an ecosystem in dire need of dignified care; or, ecological dignity.

A final common complaint is that although dignity might be a vitally important concept, its ground or normative foundation often remains uncertain and inadequately delineated (Miller, 2017). Badcott and Leget (2013), on the other hand, note that the general public seems to possess a somewhat immediate and relatively clear intuitive understanding of what might infringe upon the respect for human dignity. If intuition is all that is required, awareness of theoretical aspects of dignity and their underlying principles might not be necessary after all? However, in light of the recent attention directed toward the threat of human dignity in outdoor therapy practices, an unpacking of fundamental moral attitudes and values seem warranted. This text is therefore geared toward assisting outdoor therapy professionals and administrators alike to fully appreciate the nature and relevance of human dignity for their practice, which again is offered herein to spark an introspective look at our own practices and to identify situations where failure to treat people, and the environment for that matter, in dignified ways might arise. It is time for change, and to create new, more dignifying and caring, *stories*, we propose, where one path forward is to adopt an integrative and interconnected understanding of dignity driven by a relational ontology.

### An Integrative Understanding of Dignity

While there are various ways of understanding dignity, an integrative approach (Leget, 2013) combines three aspects: (a) an *intrinsic* dignity, which refers to the unconditional worth of every human being; (b) a *subjective* dignity, which is each person’s unique experience of dignity; and (c) social or *relational* dignity, which understands dignity as an intersubjective category that is constantly constituted and negotiated by people and circumstances. Subjective dignity is often co-determined by one’s environment, where the idea is that the more the experience of one’s dignity is undermined by one’s circumstances, the harder it is to sustain a notion of dignity. While intrinsic dignity in essence is independent from empirical reality, subjective feelings of self-worth are frequently enhanced or undermined in any given situation. An integrative view of dignity acknowledges a mutual interdependence of these three aspects; however, according to Leget (2013) relational dignity is of most relevance to ethics of care because of its sensitivity to particular situations, contexts and *complex webs of relations*. When extending this web to include environments and other species, we come closer to the interconnectedness that is relevant for outdoor therapies.

Relational reciprocity is important in the context of healthcare, in the sense that providing care that is not dignified, also jeopardizes the dignity of practitioners (Pols et al., 2018). Dignity signifies an ethical relationship between selves and others, and because of this reciprocity, dignity is not reducible to the judgment of one person alone. According to Pols et al. (2018), dignity emerges in social contexts where creating and maintaining ethical relations take place in processes of engagement, mirroring, and constant negotiations of values and attitudes. In future investigations of dignity and care in outdoor therapies, we may find that caregivers do not merely follow rules that prioritize some values over others, but rather co-labor with rules if they believe dignity is at stake.

Andorno (2013) reminds us that promotion of patients’ dignity is a crucial element of the medical profession and perhaps has become especially urgent in the often time-pressured context of modern healthcare. Critically, it is in the delivery of healthcare that respect for dignity is often noticeably threatened or compromised (Badcott and Leget, 2013). All clients are to a greater or lesser extent vulnerable. For instance, in the case of mental health care for children and adolescents, they are potentially vulnerable first to their young age and next due to the mental ill-health from which they are suffering. In addition, they may be vulnerable due to distress for instance in relation to receiving care at a clinic or the asymmetric power relationship with their assigned carer(s). If a youth chooses to take part in an outdoor therapy treatment, or happens to be forced, additional vulnerabilities can arise. Another example from the context of nature-based therapy is for instance the concern for confidentiality when client and therapist in a local park setting (as opposed to an indoor office setting) may come across someone the client knows. Predicting this possibility is a necessary responsibility of the therapist to negotiate with the client to ensure dignity in the moment it happens. These different contexts of outdoor therapies all represent demanding terrain to navigate an ethic of care in situations where dignity issues are at stake.

## In Pursuit of an Ethic of Care for Outdoor Therapies

Ethics of care originated in feminist writing of the 1980s (e.g., Gilligan, 1989) and has since evolved into a “mosaic of insights with critical potential and a great sensitivity to contextual nuance” (Leget, 2013, p. 950). Ethics of care should be sensitive to the particularity of situations rather than the generalizable features; to the subtle ways in which people may be excluded, marginalized, disrespected, or devalued. Care needs to be attuned to the complex webs of personal relations and in particular our emotional attachments and vulnerabilities. This includes human and ecological awareness and sensitivity as discussed above. A genuine interest in people’s lived experience requires an open-minded approach in which one abstains from, or minimizes, using predefined categories, pathologies and labels, and instead, adopts a resource-focused, person-centered and ecological approach. As such, care can include everything that we do to maintain, continue and repair our collective life worlds so that we can live in it as well as possible (Leget, 2013).

### Moving Forward: Proposed Steps Toward Relational-Dignified Care

“But dignity cannot only be *violated*. It can (and must) also *be positively promoted*” (Andorno, 2013, p. 972, italics in original).

Care ethics is a theoretical framework particularly well-suited to explore how remaking and advancing dignity in a less individualistic and more relational fashion might improve practice. A synergistic relationship between relational dignity and care ethics maps the interaction between ethics as caring, and ethics as dignity (Gilligan, 1989; Miller, 2017). As such, we are interested in the web of relations that helps to make meaningful the world in which our clients live (e.g., their homes, communities, local ecosystems, and other socio-economic, cultural and political realites).

We must reiterate two aspects with regards to this focus on care-in-practice. First, it does not render the one on the receiving end passive and it is not in any way meant to circumscribe the agency of those receiving care. Second, our actions as carers correspond with and display our attitudes, where respect is most often the attitude discussed in conjunction with dignity. According to Miller (2017), care can function in a similar manner, in the sense that care also embodies our attitudes. Care and respect as an attitude differs, though, and we shall dwell on this for a moment. Respect is an attitudinal recognition of another’s dignity that, in terms of action, keeps us from interfering with their lives directly. Care, in contrast, entails the attitudinal recognition of another’s dignity, but encourages the action of the carer stepping in to support the life situation of the one for whom they are caring.

By now we are well aware that not all care is good care. Good care affirms the inherent dignity of the person cared for, ideally while also supporting those in need in their agency and flourishing. Care that is insufficient or bad is most often understood as care that fails to meet the expressed or implicit needs of the care receiver. According to Miller (2017), the attitude of care is as vital to the interaction as the action itself. So what does bad care that is expressed in terms of attitude look like? A person can have their needs met, but have them met in a way that compromises their dignity, which leads Miller (2017) to emphasize that *how* we meet others’ needs is just as morally significant as *that* we meet them, thus emphasizing the relational qualities of care.

### Navigating Difficult Ethical Terrain: Positive Engagement and a Moral Compass

Engagement emerges as crucial for dignified care, particularly in situations where there are conflicts about the preferable approach, when there is no consensus, choice or compromise. The attentive engagement we are after is not achieved by enforcing rules, as this would limit the possibilities of co-laboring with our clients to find the better solution in a difficult situation, in addition to excluding the affective and introspective process that might be required. Aided by such positive engagement and use of what Pols et al. (2018) call a *moral compass*, we believe that outdoor therapy practitioners are better equipped to navigate potentially difficult terrain and provide relational and dignified care (e.g., consider the involuntary client scenario above). Such an engaging and relational stance will not guarantee perfect outcomes, nor is engagement in itself sufficient. Professional skills and supportive conditions are also needed. As such, building supportive infrastructure for personal engagement (i.e., from leadership and administration in organizations) may guarantee a general commitment to good care in frontline situations where concrete values are in tension.

### Ecological Dignity: Dignity Makers, the Pursuit of Goodness, and Care for All

Given the increased interest in relational dignity but the present lack of clarity regarding its translation into practice, it could be useful to investigate further how the concept is understood by those who have participated in outdoor therapies (e.g., Dobud, 2021). Dupré (2013) refers to the role of those whose dignity has been lost or compromised as *dignity makers*. Insights from dignity makers, or former participants, can help us elicit what an outdoor therapy practice that safeguards dignity should entail.

Furthermore, ideals such as relational-dignified care in outdoor therapy take their shape in practices amidst different notions of what is *good* to do. All participants, and the outdoor environment, play their part. By analyzing the alignments or tensions in the interaction between participants, the context, and the goods they pursue or embed, we might understand better how situations unfold in which dignity is a concern. While acknowledging that goodness is a *loose* or sensitizing concept, according to Pols et al. (2018), it is possible to study forms of good, as well as forms of *doing* good, as this is preferably part of both our socio-material world, our thinking, and our future, and could serve as pointers to identify examples of dignified care within outdoor therapies in our overall pursuit of *goodness*.

Finally, a field of practice guided by ecological dignity promotes care for all; clients, their families, communities, our colleagues in practice, and nature herself. Integrative systems thinking is necessary to envelope the broad range of actors in relationship during outdoor therapy. Good practice will then be comprised of multiple informants and feedback systems including our dignity makers, those who work alongside us, and the environments in which our programs and services are offered. To illustrate this concept, imagine taking a client group into their favorite forested area (relational dignity shown to clients), noticing degradation of the ecosystem from overuse of that site (an indignity needing to be addressed), and allowing the place time for restoration (showing care and relational dignity). We would suggest these practice behaviors be openly discussed with clients to reinforce the concepts and have a live experience of the care ethics proposed herein.

## Concluding Remarks

Considering the brevity of this paper, we concentrated on a relational view of dignity and the performative functions of care.

“*When we care well for others, we acknowledge and highlight their inherent worth and dignity. We recognize, represent, and reflect their value back to them, to ourselves, and to others who stand in social relation with them. Care that is dignifying in this way does not originate dignity in others. Dignity is inherent worth and value is already present in those cared for. Thus, good care acknowledges and preserves something that is already there*” (Miller, 2017, p. 113).

In other words, relational-dignified care is not something that is earned, or to a greater or lesser extent deserved, it is something that everyone in need of care could receive – a bit of goodness in their lives – where good care can magnify, nurture, and promote the dignity of others.

## Author Contributions

Both authors contributed toward generating ideas and revised the final manuscript.

## Conflict of Interest

The authors declare that the research was conducted in the absence of any commercial or financial relationships that could be construed as a potential conflict of interest.

## Publisher’s Note

All claims expressed in this article are solely those of the authors and do not necessarily represent those of their affiliated organizations, or those of the publisher, the editors and the reviewers. Any product that may be evaluated in this article, or claim that may be made by its manufacturer, is not guaranteed or endorsed by the publisher.
